# Better outcomes after mini-subvastus approach for primary total knee arthroplasty: a Bayesian network meta-analysis

**DOI:** 10.1007/s00590-020-02648-9

**Published:** 2020-03-10

**Authors:** Filippo Migliorini, Paolo Aretini, Arne Driessen, Yasser El Mansy, Valentin Quack, Markus Tingart, Jörg Eschweiler

**Affiliations:** 1 Department of Orthopaedics, RWTH Aachen University Clinic, Pauwelsstraße 30, 52074 Aachen, Germany; 2 Fondazione Pisana per la Scienza, Via Ferruccio Giovannini 13, 56017 Pisa, Italy; 3 Department of Orthopaedic and Traumatology, Alexandria University, Alexandria, Egypt

**Keywords:** Total knee arthroplasty, Medial parapatellar, Subvastus, Midvastus, Quadriceps sparing

## Abstract

**Introduction:**

Alternatives to the classical medial parapatellar (MPP) approach for total knee arthroplasty (TKA) include the mini-medial parapatellar (MMPP), mini-subvastus (MSV), mini-midvastus (MMV) and quadriceps-sparing (QS) approaches. The best approach has been not fully clarified. The purpose of the present study was to conduct a Bayesian network meta-analysis comparing these approaches.

**Materials and methods:**

The present analysis was carried out according to the PRISMA extension statement for reporting systematic reviews incorporating network meta-analyses of healthcare interventions. The databases search was performed in October 2019. All clinical trials comparing two or more approaches for primary TKA were considered for inclusion. The baseline comparability was evaluated through the analysis of variance (ANOVA) test. The statistical analysis was performed through the STATA software/MP. A Bayesian hierarchical random-effects model analysis was adopted in all the comparisons.

**Results:**

Data from 52 articles (4533 patients) were collected. The mean follow-up was 20.38 months. With regard to diagnosis, gender, age and BMI, adequate baseline comparability was detected. The MSV approach ranked better concerning clinical scores (the lowest visual analogic scale, the higher KSS and KSFS) and functional outcomes (the shortest straight leg raise, the greatest degree of flexion and range of motion). Concerning perioperative data, the MSV evidenced the shortest hospital stay, while the MPP the shortest surgical duration and lowest estimated blood loss.

**Conclusion:**

According to the main findings of the present study, the mini-subvastus approach for total knee arthroplasty demonstrated superior overall compared to the other approaches. Orthopaedic surgeons should consider this approach in the light of the evidence and limitations of this Bayesian network meta-analysis.

## Introduction

While the overall effectiveness of total knee arthroplasty (TKA) is largely unquestionable, the best surgical approach is still to be determined. The medial parapatellar approach, introduced by Von Langenbeck [1], is still regarded as the standard. The main benefit of this approach is that it provides the best exposure of knee surfaces. On the other hand, it has been criticized for introducing massive damage to the articular capsule, patellar and quadriceps tendons, extensor apparatus, soft tissue and vascular structures. Minimally invasive approaches to TKA have been evolved for the purpose of preserving the extensor mechanism as much as possible [2, 3]. The first minimally invasive approach for knee arthroplasty was described by Repicci and Eberle [4] for implantation of a unicompartmental prosthesis. Then came techniques extending to TKA. The mini-subvastus (MSV) approach was introduced by Hoffman et al. [5] in 1991 as a way to minimize extensor damage and preserve the vascular supply to the patella. Furthermore, this approach avoided eversion of the patella, thereby reducing the risk of tendon and cartilage damage [2, 3]. In 1997, Engh et al. [6] described the mini-midvastus (MMV) approach. In addition to the advantages of the MSV, this approach provided better exposure to all knee structures. Finally, Tria et al. [7] introduced the quadriceps-sparing (QS) approach in 2003 and Scudieri et al. the mini-medial parapatellar (MMPP) approach in 2004 [8]. The latter is a shortened version of the MPP. To our knowledge, no study has compared data from all these approaches to establish the best approach for TKA. Hence, the purpose of the present study was to perform a Bayesian network meta-analysis to compare these approaches and determine the most effective. We focused on perioperative data, clinical and functional outcomes.

## Materials and methods

### Search strategy

The present Bayesian network meta-analysis was carried out according to the PRISMA extension statement for reporting systematic reviews incorporating network meta-analyses of healthcare interventions [9]. To orient the literature search, the following features were defined:

- *P* (*population*) end-stage knee joint disease;
- *I* (*intervention*) total knee arthroplasty;
- *C* (*comparison*) MPP, MMV, MSV, QS, MMPP;
- *O* (*outcomes*) perioperative data, functional outcomes, clinical scores.

### Data source

Two authors (FM and JE) independently performed the initial search. In October 2019, the main databases were accessed: PubMed, Google Scholar, Embase and Scopus. The following keywords were used in combination: *total knee arthroplasty, total knee replacement, prosthesis, medial, quadriceps sparing, midvastus, subvastus, mini*-*medal parapatellar, KSS, KSFS, range of motion, flexion, straight leg raises, hospital duration*. All pertinent titles and abstracts were screened, and if matching the topic, the full text was accessed. Bibliographies of the included studies were also cross-referenced. Disagreements between the authors were debated and mutually solved.

### Eligibility criteria

All clinical trials comparing two or more approaches for primary TKA were considered for inclusion. According to the Oxford Centre for Evidence-Based Medicine, only articles with levels I and III evidence were considered for the present study. Articles were limited to English, German, Italian, French and Spanish. Case series, case reports, letters, expert opinions and editorials were excluded. No differences concerning the type of implants were made; only the surgical approach was pivotal for inclusion. Missing data under our outcomes of interest warranted exclusion. Disagreements were debated and mutually solved.

### Outcomes of interest

Two authors (FM and JE) independently screened all articles resulting from the search. For each approach, study generalities and patient demographics were noted: type of study, number of procedures, duration of follow-up, surgical approach(es), percentage of female and osteoarthritic (OA) patients, mean age and body mass index (BMI). Data concerning the following outcomes of interest were collected: perioperative data (duration of surgery and hospitalization, total estimated blood loss), functional outcomes (range of motion (ROM), flexion, straight leg raise (SLR) [10]) and clinical scores (visual analogic scale (VAS) for pain, the Knee Society Score (KSS) and its related function subscale (KSFS) [11]).

### Methodological quality assessment

The methodological quality of the present meta-analysis was evaluated using the PEDro appraisal score (http://www.pedro.org.au/english/downloads/pedro-scale/), which has been validated for this type of study [12]. The PEDro score is an aggregate of dichotomically assigned points given to studies based on the presence or absence of specific endpoints such as eligibility criteria, allocation, baseline comparability, blinding, follow-up, type of analysis, point estimates and variability. Values > 6 points are considered satisfactory.

### Statistical analysis

The statistical analyses were performed by the senior author (FM). The baseline comparability was evaluated through the analysis of variance (ANOVA) test. Values of *P* > 0.5 were considered satisfactory to verify comparability. The statistical analysis was performed through the STATA software/MP 14.1 (Stata Corporation, College Station, TX). A Bayesian hierarchical random-effects model analysis was adopted for all the comparisons. We referred to the generic inverse variance statistic method for continuous data analysis with standardized mean difference effect measure. The edge network plot was performed to analyse connections, contribution weights between studies, and to detect direct and indirect comparisons. To evaluate loop-specific inconsistency, heterogeneity and related inconsistency factor (IF), the if test was performed. To evaluate for overall inconsistency, the equation for global linearity via the Wald test was used. If the P value was > 0.05, the null hypothesis could not be rejected and the consistency assumption could be accepted at the overall level of each treatment. The interval plot was performed to rank the estimated effect (EE) of the endpoints between them. Confidence and percentile intervals (CI and PrI) were each set at 95%. The funnel plot was performed to assess the risk of publication bias for each comparison.

## Results

### Identification of eligible studies

**Fig. 1:**
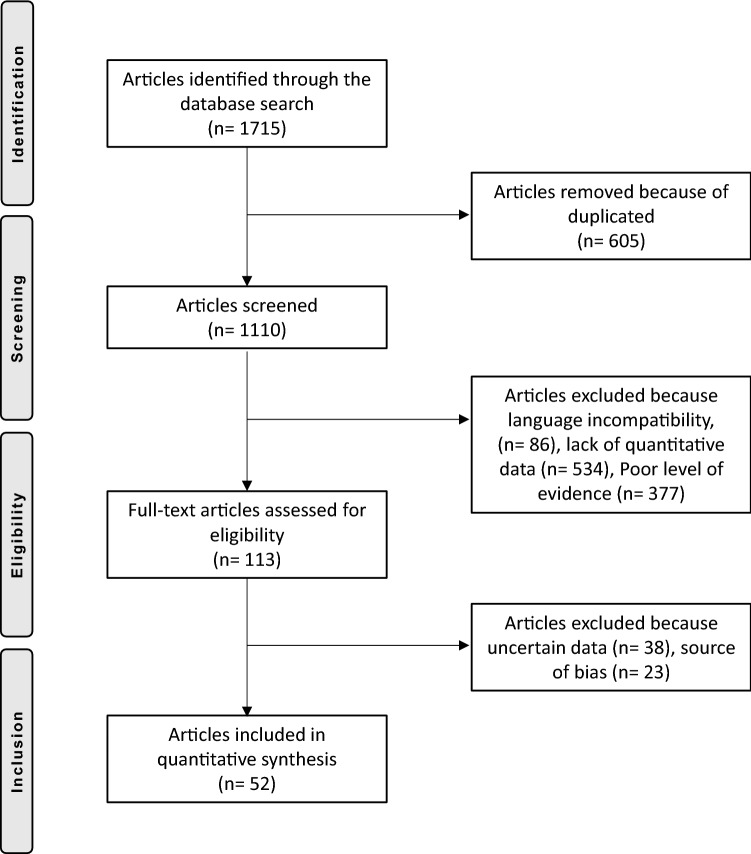
Flow chart of the literature search

A total of 1715 articles were obtained from the initial search. Of them, 605 were duplicates. A further 377 were rejected because of poor levels of evidence. Another 86 articles were excluded because of language barriers: Chinese, Polish, unknown. Another 534 articles were excluded because they did not report quantitative data under the outcomes of interest. Thirty-eight were excluded because of uncertain data or incomplete results and 23 due to source of publication bias or excessive heterogeneous results. This last operation left 52 articles for review: 34 randomized clinical trials (RCTs), ten prospective cohort studies (PCS), eight retrospective cohort studies (RCS). The flow chart of the literature search is shown in Fig. 1.

### Methodological quality assessment

All included articles stated clearly their eligibility criteria and demonstrated satisfactory baseline comparability. A random and concealed allocation was performed in 32% of the studies. 8% performed a single blinding, and 15% had blinded assessors. Only a third of the included studies performed an adequate follow-up. Almost all the articles performed an adequate analysis, and the intention to treat was satisfied. In the end, the overall PEDro score was 7.42, detecting an optimal quality for the methodological assessment. The PEDro scores across the studies are shown in Table 1.

**Table 1:** Demographic baseline of the included studies

References	Type of study	PEDro score	Knees (*n*)	Follow-up (months)	Type of approach	Knees (*n*)	OA patients (%)	Female (%)	Mean age (years)	BMI (kg/m^2^)
Aglietti et al. [13]	RCT	9	60	3.00	MSV	30		60.00	70.00	28.08
					QS	30		60.00	71.00	28.07
Aslam et al. [14]	RCT	10	84	12.00	MMV	42	100.00	30.00	68.80	30.60
					MPP	42	100.00	57.14	68.60	30.10
Avci et al. [15]	RCS	6	39	23.50	MMV	19	100.00	78.94	64.53	32.02
					MPP	20	100.00	90.00	67.25	32.56
Boerger et al. [2]	PCS	5	120	3.00	MSV	60	100.00	77.00	69.00	28.00
					MPP	60	100.00	75.00	68.00	29.00
Bridgman et al. [16]	RCT	7	224	13.00	MSV	113		48.00	70.10	
					MPP	111		49.00	70.90	
Bonutti et al. [17]	RCT	9	102	24.00	MSV	51		84.00	70.00	31.00
					MMV	51		84.00	70.00	31.00
Chalidis et al. [18]	RCT	8	100	24.00	MMV	50	100.00	92.00	70.10	34.60
					MPP	50	100.00	88.00	71.20	34.20
Chiang et al. [11]	RCT	10	75	24.00	QS	38	100.00	90.00	69.70	28.60
					MPP	37	100.00	90.00	69.80	29.60
Cho et al. [19]	RCT	8	66	12.00	MMV	33	100.00	96.00	65.50	29.10
					MPP	33	100.00	93.93	67.00	28.00
Dabboussi et al. [20]	PCS	5	80	3.00	MMV	40	100.00			
					MPP	40	100.00			
Feczko et al. [21]	RCT	7	69	6.00	MMV	36	95.00	63.88	65.14	28.26
					MPP	33	100.00	66.66	64.88	28.56
Han et al. [22]	RCT	9	30	24.00	MMPP	15	100.00		66.00	26.90
					MPP	15	100.00		64.00	26.40
Heekin et al. [23]	RCT	9	80	24.00	MMV	40		14.00	65.13	30.98
					MMPP	40		35.00	65.13	30.98
Hernandez-Vaquero et al. [24]	RCT	7	62	6.00	MMV	26	100.00	80.77	70.80	32.10
					MPP	36	100.00	80.33	70.50	30.80
Huang et al. [25]	RCS	6	96	60.00	MMPP	35	100.00	85.71	69.20	27.00
					QS	31	100.00	93.55	69.30	26.90
					MPP	30	100.00	93.33	71.20	26.70
Jarvis et al. [26]	RCT	6	53	6.00	MSV	27	100.00	87.00	69.00	31.40
					MMV	26	100.00	61.54	74.20	29.80
Jung et al. [27]	PCS	6	40	58.40	MSV	21				
					MPP	19				
Juosponis et al. [28]	RCT	8	70	3.00	MMV	35	100.00	85.71	72.00	27.95
					MPP	35	100.00	85.71	71.40	29.08
Karachalios et al. [29]	RCT	8	100	23.00	MMV	50	92.00	62.00	71.10	32.00
					MPP	50	92.00	70.00	70.80	31.50
Karpman et al. [30]	RCT	8	59	6.00	MMV	20	100.00	65.00	74.00	30.00
					QS	20	100.00	60.00	73.00	28.00
					MPP	19	100.00	53.00	73.00	29.00
Kim [31]	RCT	10	50	12.00	MMV	23	100.00		67.00	27.10
					MPP	22	100.00		68.00	28.40
King et al. [32]	RCS	5	150	1.50	QS	100	95.00	52.00	67.00	30.00
					MPP	50	90.00	66.00	28.00	32.00
Laskin et al. [3]	RCS	5	58	3.00	MMV	26			70.00	30.00
					MPP	26			68.00	29.00
Li et al. [33]	RCT	8	50	12.00	MSV	25	100.00	64.00	69.90	25.80
					MPP	25	100.00	64.00	68.10	25.50
Liebensteiner et al. [34]	PCS	5	38	2.00	MMV	19		57.89	66.70	30.20
					MPP	19		52.63	67.60	31.50
Lin et al. [35]	RCT	9	80	2.00	QS	40	100.00	67.50	69.60	28.10
					MMPP	40	100.00	67.50	70.20	29.00
Lin et al. [36]	RCT	9	100	24.00	QS	35	100.00	86.00	67.70	26.30
					QS	30	100.00	87.00	70.00	25.90
					MMPP	35	100.00	86.00	68.50	25.90
Liu et al. [37]	RCT	8	90	24.00	MMV	45				
					MPP	45				
Maru et al. [38]	PCS	6	77	3.00	MMV	37	100.00	51.35	71.00	
					MPP	40	96.00	62.50	70.00	
McAllister et al. [39]	PCS	7	180	13.00	MMPP	91	100.00	51.64	63.00	
					MPP	89	100.00	49.43	63.00	
Mehta et al. [40]	RCT	7	55	6.00	MSV/MMV	26		73.17	59.80	
					MPP	29		73.17	61.40	
Mukherjee et al. [41]	RCT	8	100		MMV	20				
					MPP	80				
Nestor et al. [42]	RCT	10	54	3.00	MMV	27		66.66	66.70	29.60
					MPP	27		66.66	66.70	29.60
Nutton et al. [43]	RCT	10	23	6.00	MMV	11	100.00	33.00	73.00	31.10
					MPP	12	100.00	50.00	70.00	29.30
Pescador et al. [44]	RCT	8	96	96.00	MSV	45		68.88	71.20	28.40
					MPP	51		72.55	69.30	27.90
Pongcharoen et al. [45]	RCT	8	60	12.00	MMV	30	100.00	83.33	67.00	27.00
					MPP	30	100.00	69.69	67.00	26.00
Rahman et al. [46]	RCS	5	120	3.00	MMPP	60	100.00	75.00	59.80	
					MPP	60	100.00	77.00	62.00	
Schroer et al. [47]	PCS	6	300	24.00	QS	150		62.00	71.00	31.00
					MPP	150		61.00	70.00	32.00
Seon et al. [48]	PCS	6	84	12.00	MMV	41	100.00	80.48	64.20	
					MPP	43	100.00	76.74	64.20	
Dayton et al. [49], Stevens-Lapsley et al. [50]	RCT	8	41	3.00	MMPP	22	100.00	54.00	64.60	30.50
					MPP	19		45.00	64.00	31.30
Tasker et al. [51]	RCT	8	83	24.00	MMV/MSV	40	45.00	63.00	67.30	
					MPP	43	99.00	63.00	68.20	
Tenholder et al. [52]	RCS	6	118		MMPP	69		56.00	66.80	29.30
					MPP	49		46.94	63.50	31.50
Thienpont et al. [53]	RCT	8	300	24.00	MMPP	150	100.00	66.66	68.00	30.40
					MPP	150	100.00	70.00	69.00	29.8
Tsuji et al. [54]	PCS	5	20	0.50	MMV	10	100.00	60.00	68.40	28.10
					MPP	10	100.00	80.00	69.80	28.90
Unnanuntana et al. [55]	RCS	6	64	60.00	MMPP	31				
					MPP	57				
Unwin et al. [56]	RCT	8	66	72.00	MMV/MSV	32		75.75	67.00	
					MPP	34		75.75	67.00	
Varela-Egocheaga et al. [57]	RCT	8	100	36.00	MSV	50		72.00	68.02	30.97
					MPP	50		74.00	70.64	30.62
Watanabe et al. [58]	RCS	6	48	48.00	MMV	25	84.00	80.00	71.00	28.10
					MPP	23	78.00	73.91	71.00	26.30
Wegrzyn et al. [59]	RCT	10	36	2.00	MSV	18	100.00	72.00	67.00	30.00
					MPP	18	100.00	72.00	64.00	31.00
Wülker et al. [60]	RCT	8	134	12.00	MSV	66	92.00	72.70	70.20	29.30
					MPP	68	88.00	70.10	29.30	
Zhang et al. [61]	RCT	8	82	12.00	MPP	40		54.00	65.90	26.20
					MMPP	42		50.00	64.50	24.90
Zhu et al. [62]	PCS	6	67	109.20	MMPP	30		93.30	67.90	27.60
					MPP	37		83.80	65.30	27.70

*MPP* medial parapatellar, *MMPP* mini-medial parapatellar, *QS* quadriceps sparing, *MMV* mini-midvastus, *MSV* mini-subvastus

### Patient demographics

Data from 4533 patients were collected. The mean follow-up was 20.38 (range 3 to 109) months. In the MVV group, a total of 880 patients were analysed. 96% suffered from OA, and 67% were female. Their mean age was 68.64 ± 3.5 years, and mean BMI was 29.46 ± 1.7 kg/m^2^. In the MPP group, a total of 2026 patients were analysed. 98% suffered from OA, and 69% were female. The mean age was 66.27 ± 7.9 years, and mean BMI was 29.29 ± 1.9 kg/m^2^. In the MSV group, a total of 604 patients were analysed. 90% suffered from OA, and 70% were female. The mean age was 67.72 ± 3.1 years, and the mean BMI was 29.61 ± 1.5 kg/m^2^. In the MMPP group, a total of 660 patients were analysed. 100% suffered from OA, and 66% were female. The mean age was 66.31 ± 2.5 years, and mean BMI was 28.00 ± 2.4 kg/m^2^. In the QS group, a total of 474 patients were analysed. 99% suffered from OA, and 73% were female. The mean age was 70.13 ± 1.9 years, and mean BMI was 29.03 ± 1.4 kg/m^2^. Among the studies, with regard to diagnosis, gender, age and BMI, adequate baseline comparability was detected (*P* = 0.7, *P* = 0.8, *P* = 0.9, *P* = 0.8, respectively). Patient demographics for the included studies are shown in Table 1.

### Outcomes of interest

**Fig. 2:**
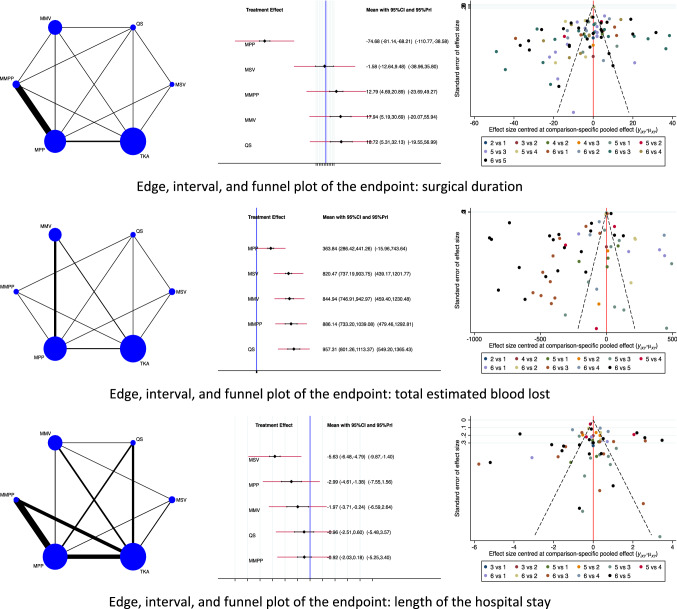
Results of the network comparison perioperative data

Concerning perioperative data, the MSV approach demonstrated the lowest duration of hospitalization (EE: − 5.63, 95% CI: − 6.48 to − 4.79), followed by the MPP approach (EE: − 2.99, 95% CI: − 4.61 to − 1.38). The MMPP approach reported the highest duration of hospitalization (EE: − 0.92, 95% CI: − 2.03 to 0.18). The test for overall inconsistency scored *P* = 0.2. The MPP approach demonstrated the lowest value of total estimated blood loss (EE: 363.84, 95% CI: 286.42 to 441.26) followed by the MSV approach (EE: 820.47, 95% CI: 737.19 to 903.75). The QS approach reported the greatest value of total estimated blood loss (EE: 957.31, 95% CI: 801.26 to 1113.37). The test for overall inconsistency scored *P* = 0.9. The MPP approach demonstrated the shortest surgical duration (EE: − 74.68, 95% CI: − 81.14 to − 68.21) followed by the MPP approach (EE: − 1.58, 95% CI: − 12.64 to 9.48). The QS approach reported the longest surgical duration (EE: 18.72, 95% CI: 5.31 to 32.13). The test for overall inconsistency scored *P* = 0.9. The network results concerning the endpoint perioperative data are shown in Fig. 2.

**Fig. 3:**
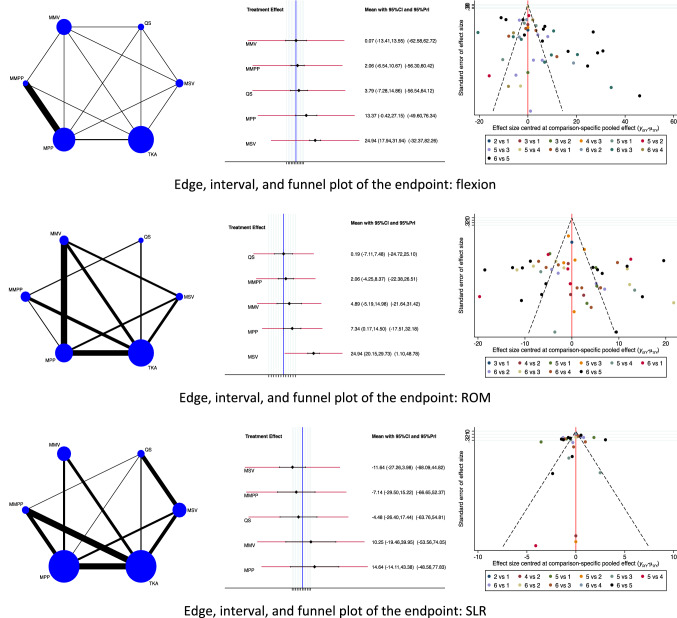
Results of the network comparison functional outcomes

Regarding the functional outcomes, the MSV approach detected the greatest degree of flexion (EE: 24.94, 95% CI: 17.94 to 31.94) followed by the MPP approach (EE: 13.37, 95% CI: − 0.42 to 27.15). The MMV approach reported the lowest degree of flexion (EE: 0.07, 95% CI: − 13.41 to 13.55). The test for overall inconsistency scored *P* = 0.3. The MSV approach detected the greatest ROM (EE: 24.94, 95% CI: 20.15 to 29.73) followed by the MPP approach (EE: 7.34, 95% CI: 0.17 to 14.50). The QS approach reported the lowest ROM (EE: 0.19, 95% CI: − 7.11 to 7.48). The test for overall inconsistency scored *P* = 0.5. The MSV approach demonstrated the shortest SLR (EE: − 11.64, 95% CI: − 27.26 to 3.98) followed by the MMPP approach (EE: − 7.14, 95% CI: − 29.50 to 15.22). The MPP approach reported the longest SLR (EE: 14.64, 95% CI: − 14.11 to 43.38). The test for overall inconsistency scored *P* = 0.6. The network results concerning the endpoint functional outcomes are shown in Fig. 3.

**Fig. 4:**
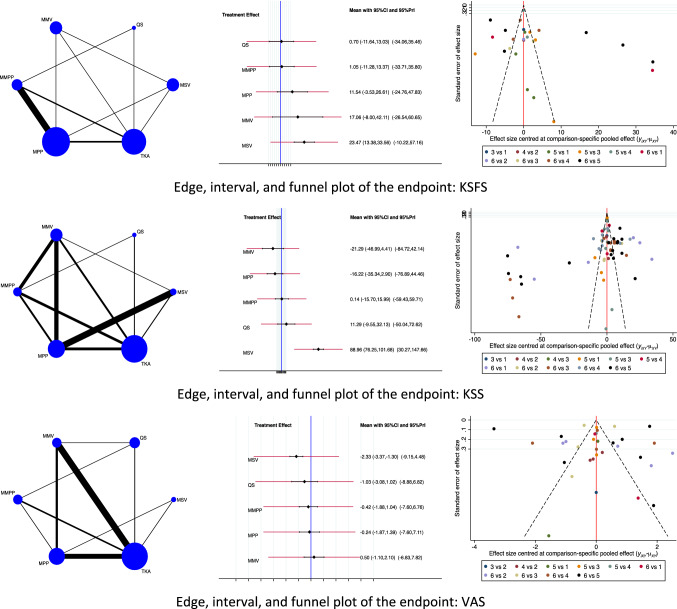
Results of the network comparison clinical scores

Concerning the clinical scores, the MSV approach demonstrated the highest KSFS scores (EE: 23.47, 95% CI: 13.38 to 33.56) followed by the MMV approach (EE: 17.06, 95% CI: − 8.00 to 42.11). The QS approach reported the lowest value of KSFS (EE: 0.70, 95% CI: − 11.64 to 13.03). The test for overall inconsistency scored *P* = 0.7. The MSV approach demonstrated the highest KSS scores (EE: 88.96, 95% CI: 76.25 to 101.68) followed by the QS approach (EE: 11.29, 95% CI: − 9.55 to 32.13). The MMV approach reported the lowest KSS scores (EE: − 21.29, 95% CI: − 46.99 to 4.41). The test for overall inconsistency scored *P* = 0.4. The MSV approach demonstrated the lowest VAS pain score (EE: − 2.33, 95% CI: − 3.37 to − 2.30) followed by the QS approach (EE: − 1.03, 95% CI: − 3.08 to 1.02). The MMV approach reported the highest value of VAS (EE: 0.50, 95% CI: − 1.10 to 2.10). The test for overall inconsistency scored *P* = 0.05. The network results concerning the endpoint clinical scores are shown in Fig. 4.

## Discussion

The main results of this Bayesian network meta-analysis encourage to perform the minimally invasive subvastus approach for total knee arthroplasty. Concerning clinical scores, functional outcomes and length of the hospital stay, the MSV approach outperformed all other approaches. Surgical duration and total estimated blood loss were lower in the MPP approach. The transitivity between studies was always satisfied, and the equation for global linearity via the Wald test found no statistically significant inconsistency among the studies, attesting reliability of the present results.

The intention of the minimally invasive approaches is to provide quicker recovery after TKA by preventing damage to the extensor mechanism. However, the MPP demonstrated a very short length of the hospital stay compared to the other minimally invasive techniques. Regarding perioperative outcomes, the QS approach reported the longest surgical duration and the highest value of total estimated blood loss. This can be explained by the reduced joint exposure of minimally invasive surgeries compared with the traditional approach. Peersman et al. [63], in a retrospective cohort study including 6489 patients, found correlation between an increased surgical duration and an augmented risk of surgical site infection during TKA. Reduced visibility can considerably complicate component installation, prolong the learning curve and generate skin sloughs [52, 64]. The QS approach especially requires more attention to retractor positioning, since they damage bones (particularly when osteoporotic) and soft tissue [13, 30]. The advantage of the QS approach is that it can be easily converted to MMPP or MPP [13].

Concerning clinical scores (KSS, KSFS, VAS), the MSV approach performed better overall. The equation for global linearity via the Wald test evidenced no statistically significant inconsistency among the studies. Thus, the assumption of transitivity can be accepted. The MMV reported the lowest value of KSS and VAS, while the QS the lowest KSFS. Concerning functional outcomes (SLR, ROM, flexion), the MSV approach performed better overall. No statistically significant inconsistency was found through the equation for global linearity via the Wald test; therefore, transitivity between the studies is assumed. As expected, the MPP evidenced the longest SLR, while the MMV the lowest degree of flexion and the QS the lowest ROM.

One of the purposes of minimally invasive TKA is to reduce damage to the quadriceps tendon, in order to guarantee quicker recovery of the extensor mechanism function. The straight leg raise (SLR) is used to assess quadriceps restoration after TKA [65, 66]. SLR times were longer in the traditional MPP group and strongly improved in the other approaches, especially in the MSV approach, confirming reduced damage to the extensor mechanism in these approaches. Several studies have tried to quantify quadriceps destruction in minimally invasive TKA versus the traditional MPP using levels biomarkers indicative of muscle damage (e.g. creatine kinase, interleukin-6, myoglobin). However, their results are contrasting and controversial [67–69].

Comparing the traditional MPP to the other approaches for TKA, we highlight multiple advantages and disadvantages. First, all the other approaches provide a minimally invasive surgery. Second, they aim to preserve patellar vascularization [68], which can reduce the occurrence of patellar fractures, avascular necrosis, subluxations, dislocations, component loosening and rates of anterior knee pain [5, 7]. Third, they are supposed to promote quicker recovery of the quadriceps function and decreased post-operative pain [70, 71]. The MSV, along with the MMV, preserves the vastus medialis insertion [47, 72] and potentially reduces the risk of VMO denervation [71, 73]. Pagnano et al. [74] in a cadaveric study demonstrated that the VMO tendon inserts mostly down to the mild pole of the patella, thus proving that the MSV is the only approach able to preserve the VMO insertion on the patella. Furthermore, the MSV proximally avoids an incision to the descending genicular artery branches (musculoarticular branch) [75, 76]. Fourth, the MSV and MMV almost never require a lateral release [72, 77]. Lateral retinacular release during TKA is not fully understood. It is meant to improve patellar tracking, but can also damage patellar vascularization and reduce joint stability [78, 79]. However, due to difficult execution, a longer learning curve and the need for special instruments, it has not been very popular [13, 80]. The difficulty of execution can result in ligament–patellar maltracking, increased rates of polyethylene wear, loosening, imbalance and instability [81, 82]. To assist the surgeon, the use of a mobile window can facilitate exposure of knee surfaces and dedicated instrumentation should be considered [28, 42]. Not surprisingly, previous studies confirm that the MPP exposure is related to an optimal component positioning [35, 83, 84]. This has discouraged many surgeons from performing minimally invasive TKAs, and the MPP remains the most common approach for TKA. Indeed, our results evidenced that the MPP exposure required less surgical duration and lower estimated blood loss. However, we hypothesize that these results are strongly influenced by the learning curve.

This Bayesian network meta-analysis has several limitations. As previously mentioned, due to reduced visibility and augmented difficulties of installation, minimally invasive TKAs can result in implant malposition. Notwithstanding, implant positioning has not been evaluated, thus representing an important limitation of this study. Implant malposition relates to instability, loosening and consequent joint failure. Further studies should clarify this important endpoint. Another notable limitation of the present study is its lack of analysis for complications. This is due to a lack of data in the included studies under these endpoints. Further studies should implement analyses of complications and evaluate the feasibility of minimally invasive surgeries, especially when it comes to obese patients and patients with previous knee surgeries (e.g. high tibial osteotomies). Points of strength in this Bayesian network meta-analysis are the comprehensive nature of the literature search and the optimal baseline comparability, along with the high number of enrolled studies. To the best of our knowledge, this study represents the first study comparing multiple surgical approaches for TKA. Data from the present network analysis provide evidence in favour of the mini-subvastus approach for total knee arthroplasty. However, the present study represents a data statistical elaboration and, therefore, must be interpreted with caution.

## Conclusion

According to the main findings of the present study, the mini-subvastus approach for total knee arthroplasty demonstrated superior overall compared to the other approaches. Orthopaedic surgeons should consider this approach in the light of the evidence and limitations of this Bayesian network meta-analysis.
