# Correction to: Better outcomes after mini‑subvastus approach for primary total knee arthroplasty: a Bayesian network meta‑analysis

**DOI:** 10.1007/s00590-021-03026-9

**Published:** 2021-06-14

**Authors:** Filippo Migliorini, Paolo Aretini, Arne Driessen, Yasser El Mansy, Valentin Quack, Markus Tingart, Jörg Eschweiler

**Affiliations:** 1 Department of Orthopaedics, RWTH Aachen University Clinic, Pauwelsstraße 30, 52074 Aachen, Germany; 2 Fondazione Pisana per la Scienza, Via Ferruccio Giovannini 13, 56017 Pisa, Italy; 3 Department of Orthopaedic and Traumatology, Alexandria University, Alexandria, Egypt

## Correction to: European Journal of Orthopaedic Surgery & Traumatology (2020) 30:979–992 https://doi.org/10.1007/s00590-020-02648-9

The article Better outcomes after mini‑subvastus approach for primary total knee arthroplasty: a Bayesian network meta‑analysis, written by Filippo Migliorini, Paolo Aretini, Arne Driessen, Yasser El Mansy, Valentin Quack, Markus Tingart and Jörg Eschweiler, was originally published Online First without Open Access. After publication in volume 30, issue 6, page 979–992 the author decided to opt for Open Choice and to make the article an Open Access publication. Therefore, the copyright of the article has been changed to © The Author(s) 2021 and this article is licensed under a Creative Commons Attribution 4.0 International License, which permits use, sharing, adaptation, distribution and reproduction in any medium or format, as long as you give appropriate credit to the original author(s) and the source, provide a link to the Creative Commons licence, and indicate if changes were made. The images or other third party material in this article are included in the article’s Creative Commons licence, unless indicated otherwise in a credit line to the material. If material is not included in the article’s Creative Commons licence and your intended use is not permitted by statutory regulation or exceeds the permitted use, you will need to obtain permission directly from the copyright holder. To view a copy of this licence, visit http://creativecommons.org/licenses/by/4.0/.

The original article has been corrected.

